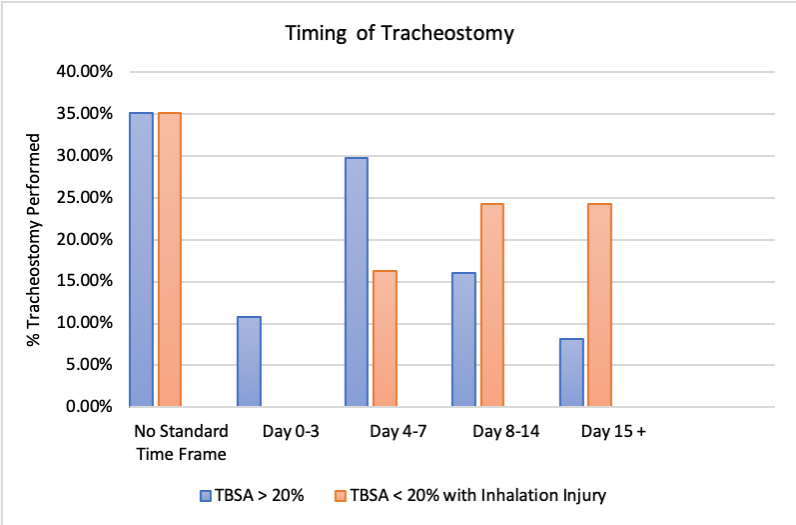# 501 The Role of a Tracheostomy in the Critically Ill Burn Patient

**DOI:** 10.1093/jbcr/irad045.098

**Published:** 2023-05-15

**Authors:** Syed Saquib, Lana Jesic, Joseph Carroll, Carmen Flores, Paul Chestovich, Douglas Fraser

**Affiliations:** Kirk Kerkorian School of Medicine at UNLV/UMC Lions Burn Care Center, Las Vegas, Nevada; Kirk Kerkorian School of Medicine at UNLV, Las Vegas, Nevada; Kirk Kerkorian School of Medicine at UNLV, las vegas, Nevada; Kirk Kerkorian School of Medicine at UNLV, Las Vegas, Nevada; Kirk Kerkorian School of Medicine at UNLV, Las Vegas, Nevada; Kirk Kerkorian School of Medicine at UNLV, Las Vegas, Nevada; Kirk Kerkorian School of Medicine at UNLV/UMC Lions Burn Care Center, Las Vegas, Nevada; Kirk Kerkorian School of Medicine at UNLV, Las Vegas, Nevada; Kirk Kerkorian School of Medicine at UNLV, las vegas, Nevada; Kirk Kerkorian School of Medicine at UNLV, Las Vegas, Nevada; Kirk Kerkorian School of Medicine at UNLV, Las Vegas, Nevada; Kirk Kerkorian School of Medicine at UNLV, Las Vegas, Nevada; Kirk Kerkorian School of Medicine at UNLV/UMC Lions Burn Care Center, Las Vegas, Nevada; Kirk Kerkorian School of Medicine at UNLV, Las Vegas, Nevada; Kirk Kerkorian School of Medicine at UNLV, las vegas, Nevada; Kirk Kerkorian School of Medicine at UNLV, Las Vegas, Nevada; Kirk Kerkorian School of Medicine at UNLV, Las Vegas, Nevada; Kirk Kerkorian School of Medicine at UNLV, Las Vegas, Nevada; Kirk Kerkorian School of Medicine at UNLV/UMC Lions Burn Care Center, Las Vegas, Nevada; Kirk Kerkorian School of Medicine at UNLV, Las Vegas, Nevada; Kirk Kerkorian School of Medicine at UNLV, las vegas, Nevada; Kirk Kerkorian School of Medicine at UNLV, Las Vegas, Nevada; Kirk Kerkorian School of Medicine at UNLV, Las Vegas, Nevada; Kirk Kerkorian School of Medicine at UNLV, Las Vegas, Nevada; Kirk Kerkorian School of Medicine at UNLV/UMC Lions Burn Care Center, Las Vegas, Nevada; Kirk Kerkorian School of Medicine at UNLV, Las Vegas, Nevada; Kirk Kerkorian School of Medicine at UNLV, las vegas, Nevada; Kirk Kerkorian School of Medicine at UNLV, Las Vegas, Nevada; Kirk Kerkorian School of Medicine at UNLV, Las Vegas, Nevada; Kirk Kerkorian School of Medicine at UNLV, Las Vegas, Nevada; Kirk Kerkorian School of Medicine at UNLV/UMC Lions Burn Care Center, Las Vegas, Nevada; Kirk Kerkorian School of Medicine at UNLV, Las Vegas, Nevada; Kirk Kerkorian School of Medicine at UNLV, las vegas, Nevada; Kirk Kerkorian School of Medicine at UNLV, Las Vegas, Nevada; Kirk Kerkorian School of Medicine at UNLV, Las Vegas, Nevada; Kirk Kerkorian School of Medicine at UNLV, Las Vegas, Nevada

## Abstract

**Introduction:**

Tracheostomy is a commonly performed procedure in critically ill patients requiring prolonged mechanical ventilation. However, nationwide practice patterns for the role of a tracheostomy in critically ill burn patients have not been well described.

**Methods:**

A 25-question Qualtrics online survey was distributed by the American Burn Association (ABA) to their physician members. Questions pertained to practitioner demographics, indications, type of tracheostomy performed, and timing of procedure. The questionnaire further ascertained how presence of inhalation injury and Total Body Surface Area (TBSA) influence timing of tracheostomy; if concurrent percutaneous endoscopic gastrostomy (PEG) tubes were performed, and whether enteral feeds were held prior to tracheostomy.

**Results:**

Thirty-seven surgeons responded to the survey and worked primarily at ABA verified academic burn centers. Open tracheostomy was performed more frequently than percutaneous tracheostomy (73% vs 27%). Eighty-three percent of surgeons deferred concomitant PEG despite healthy overlying skin due to the belief that a patient would eventually pass a swallow evaluation (74%). Tube feeds were routinely held by 67% of surgeons, most commonly 6 or 8 hours prior. The most common indication for tracheostomy was prolonged mechanical ventilation. Fifty-four percent of surgeons routinely perform a tracheostomy through open uninfected 2nd degree neck burns. As for 3rd degree neck burns, 35% will perform a tracheostomy only after excision and grafting. For patients requiring prolonged mechanical ventilation 35% of surgeons had no standard time frame for tracheostomy regardless of TBSA. For mechanically ventilated patients with >20% TBSA burns (excluding neck burns), 40% performed early tracheostomy (0-7 days) and 24% performed delayed tracheostomy (8+days). For ventilated patients with inhalational injuries and TBSA < 20%, 35% had no standard time frame, 16% performed early tracheostomy and 49% performed delayed tracheostomy.

**Conclusions:**

A national tendency toward open tracheostomy, deferring concomitant PEG, and holding tube feeds 6-8 hours prior to tracheostomy was identified. Patients with TBSA >20% seem to undergo earlier tracheostomy more frequently, whereas those with TBSA < 20% with inhalational injury more frequently undergo delayed tracheostomy. Although TBSA appears to impact timing of tracheostomy, evidence based guidelines are lacking to guide clinical practice and additional studies are needed.

**Applicability of Research to Practice:**

Survey results infer practice patterns favoring consideration toward early tracheostomy for patients with >20% TBSA burns requiring prolonged mechanical ventilation.